# Short-Term Bixin Supplementation of Healthy Subjects Decreases the Susceptibility of LDL to Cu^2+^-Induced Oxidation *Ex Vivo*

**DOI:** 10.1155/2019/9407069

**Published:** 2019-03-03

**Authors:** Lisiane Conte, Sabrina Somacal, Sabrina Marafiga Nichelle, Cristine Rampelotto, Silvino Sasso Robalo, Miguel Roehrs, Tatiana Emanuelli

**Affiliations:** ^1^Graduate Program on Pharmacology, Centre of Health Sciences, Federal University of Santa Maria, 97105-900 Santa Maria, RS, Brazil; ^2^Integrated Centre for Laboratory Analysis Development (NIDAL), Department of Food Technology and Science, Centre of Rural Sciences, Federal University of Santa Maria, 97105-900 Santa Maria, RS, Brazil; ^3^Department of Animal Science, Federal University of Santa Maria, 97105-900 Santa Maria, RS, Brazil

## Abstract

Lycopene-based medications and supplements have been developed to prevent atherosclerosis, primarily because of their ability to decrease low-density lipoprotein (LDL) oxidation. Bixin and norbixin are carotenoids found in the seeds of annatto (*Bixa orellana*) and are colorants widely used by the food industry. Some studies have already demonstrated that these compounds have antioxidant and antiatherogenic potential *in vitro* and in animal models, but there is no evidence supporting the effects of their long-term or short-term consumption by humans. The aim of this study was to evaluate the effects of short-term intake of annatto carotenoids on biochemical and oxidative stress biomarkers as well as on the susceptibility of LDL oxidation in healthy individuals, using lycopene as a positive control. The effect of daily supplementation (0.05 mg/kg of body weight (b.w.)) with bixin, norbixin, lycopene, or placebo for 7 days was evaluated in a randomized, controlled crossover study in 16 healthy volunteers (8 men and 8 women). The susceptibility of LDL to Cu^2+^-induced oxidation *ex vivo*, biochemical parameters, and oxidative stress biomarkers were evaluated. No treatment affected biochemical parameters or most oxidative stress biomarkers. However, bixin reduced the oxidation rate of the LDL lipid moiety (−275%, *p* < 0.1) and nitric oxide metabolites (NOx) (−460%, *p* < 0.1), compared to the placebo group. Moreover, we observed that the changes in these parameters were positively associated, supporting the hypothesis that bixin decreases the susceptibility of LDL to Cu^2+^-induced oxidation by decreasing NOx levels, probably by downregulating the inducible nitric oxide synthase.

## 1. Introduction

The oxidation of low-density lipoprotein (LDL) is a key event in the progression of atherosclerosis, which plays a major role in the most prevalent types of cardiovascular diseases [1]. Hypercholesterolemia, hypertension-triggered shear stress, and endothelial dysfunction favor the entrance of LDL particles in the tunica intima of blood vessels, where they are oxidized and taken up by macrophage cells leading to foam cell formation [1]. The parallel release of proinflammatory cytokines, growing factors, and metalloproteinases by macrophages promotes cell recruitment, angiogenesis, apoptosis, and degradation of the extracellular matrix [2].

Nitric oxide (NO) synthesized by endothelial nitric oxide synthase (eNOS) plays an important role in vascular homeostasis by promoting vasodilatation and inhibiting inflammation, platelet aggregation, oxidative stress, and the migration and proliferation of vascular smooth muscle cells (SMC) [3]. In contrast to eNOS, the inducible isoform of NOS (iNOS) exhibits proatherosclerotic effects [4]. The overproduction of NO by iNOS inhibits eNOS activity, impairs the activation of endothelial receptors, reduces SMC response to NO, and leads to peroxynitrite (ONOO^−^) formation after reaction with the superoxide anion radical (O_2_^−•^) [4].

Due to its highly reactive nature, ONOO- triggers lipid and LDL oxidation [5] beyond promoting oxidative changes in proteins and nucleic acids [6]. Beyond their role in oxidative stress damaging mechanisms, some oxidative biomarkers, such as advanced oxidation protein products (AOPP), are markers of protein oxidative damage, which can be produced after the modification of plasma albumin and has been shown to be implicated in the pathogenesis of atherosclerosis [7] by the amplification of proinflammatory pathways [8]. Endogenous antioxidant enzymes play a key role to control the production of reactive species as peroxides and O_2_^−•^, but we have no enzyme to remove ONOO^−^ [9]. Nonenzymatic defenses comprising endogenous small molecules, such as uric acid, glutathione, and coenzyme Q, and dietary antioxidants (vitamins, polyphenols, and carotenoids) also help to keep the homeostasis between the synthesis and removal of reactive species (RS) [6].

Dietary antioxidants play an important role in the prevention of cardiovascular diseases, where carotenoids have a distinguished place [10–12]. Lycopene, which is found at high concentrations in tomatoes, has a high antioxidant capacity and is among the most studied carotenoids. Despite several studies showing that lycopene consumption presents positive effects in cardiovascular function, its protective effect against the oxidation of LDL remains controversial [13, 14]. Thus, other carotenoids have been studied in more recent years. Bixin and norbixin are apocarotenoids found in food colorants extracted from annatto seeds (*Bixa orellana*), where they are synthetized from a lycopene molecule [15]. Despite their safety as food colorants [16], the beneficial biological effects of bixin and norbixin have been studied just in the last few years. Bixin has been shown to increase the activity of antioxidant enzymes in diabetic rats [17] and reduce the inflammatory response and atherosclerotic plaques in hypercholesterolemic rabbits [18]. Despite the promising data obtained in animal studies, there is only one human study investigating the effects of annatto carotenoids in humans [19]. In this study, a single dose of norbixin reduced the postprandial levels of oxidized LDL (oxLDL), thiobarbituric acid reactive substances (TBARS), and the levels of proinflammatory cytokines after the consumption of a hypercaloric meal by healthy volunteers [19]. However, the biological effects of multiple doses of bixin and norbixin have not been investigated in humans.

In the present study, we investigated the effects of short-term bixin and norbixin supplementation on biochemical parameters, oxidative stress biomarkers, and LDL susceptibility to oxidation *ex vivo* in healthy subjects. The effects were compared to lycopene supplementation as a positive standard as this carotenoid is considered to have cardiovascular benefits.

## 2. Materials and Methods

### 2.1. Subjects

**Figure 1:**
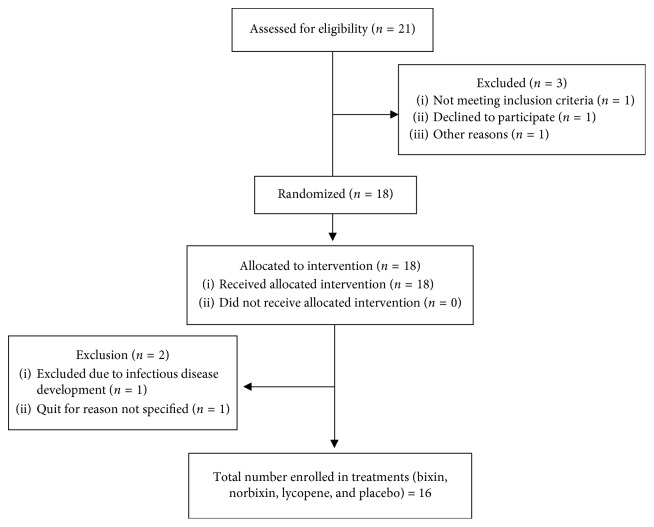
Consort flow diagram of recruitment, allocation, randomization, and retention of volunteers.

Sixteen healthy subjects (eight men and eight women; 25.4 ± 0.8 years of age, 66.5 ± 2.2 kg body weight, 1.71 ± 0.03 m body height, and 22.6 ± 0.7 kg/m^2^ body mass index) completed this randomized, double-blind, placebo-controlled crossover clinical trial. Undergraduate and graduate students aged between 18 and 35 years, with blood pressure, anthropometric measurements (weight and body mass index), and biochemical parameters (glucose, lipid profile, transaminases, urea, and creatinine) within normal values, were recruited from Federal University of Santa Maria and gave their written consent to participate in the study (Figure 1). The study was conducted according to Declaration of Helsinki, and the study protocol was approved by the local ethics committee (CAAE number: 68801917.0.0000.5346). Volunteers with alcohol/cigarette/drug addiction, dyslipidemia, diabetes, hypertension, cancer, and recent inflammatory and infectious diseases were excluded from the study. Participants that were using medications, except oral contraceptives, were also excluded.

### 2.2. Study Protocol

**Figure 2:**
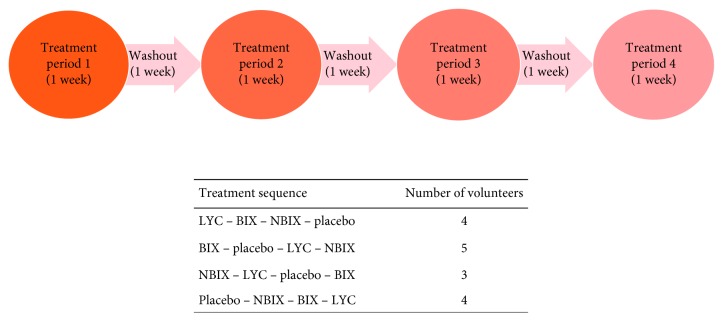
Representative figure of treatment sequence followed by volunteers.

The health status of the participants was assessed at entry, by a questionnaire (initial screening), anthropometric measurements, and blood biochemical analysis (glucose, lipid profile, alanine aminotransferase (ALT), aspartate aminotransferase (AST), urea, and creatinine). Healthy subjects were included in the study and instructed to follow their usual diet, avoiding excessive or unusual consumption of alcoholic beverages, fatty foods, annatto/carotenoid-containing food, or another unusual food during the study. The intervention period comprised one to three weeks of the washout period, followed by one week of treatment. All subjects received the four treatments during the study in different periods, resulting in 2 months of participation. Each treatment was composed of a daily capsule of placebo or 0.05 mg/kg of body weight (b.w.) of bixin, norbixin, or lycopene. Participants received seven capsules per treatment, corresponding to one week of treatment, and were instructed to take one capsule per day, preferably at breakfast. A Latin square crossover design was used. Four treatment sequences were chosen to yield a balanced design that was uniform both within periods and within sequences, and each treatment preceded every other treatment only once (Figure 2). Treatments were coded (A, B, C, and D), and sequences were randomly distributed among volunteers (Figure 2). Both volunteers and analysts had no knowledge of drawn sequences.

The dose of carotenoids chosen for this clinical trial (0.05 mg/kg b.w.) is 10 times lower than the acceptable daily intake (ADI) of lycopene (0.5 mg/kg b.w.), which was the carotenoid that had the lowest ADI value compared to bixin and norbixin (ADI: 6 and 0.3 mg/kg b.w., respectively) [20, 21]. This dose is lower than recently used by our group for a single dose study of annatto carotenoids in humans (0.06 and 1.2 mg/kg b.w. for norbixin and bixin, respectively) [19] since we would be assessing the effect of multiple doses.

Two 12 h fasting blood collections (30 mL) were performed for each treatment: at baseline (Day 0) and one day after consumption of the last capsule (Day 7). Blood was collected from the basilic or median cubital vein puncture using vacuum tubes-containing EDTA for the collection of plasma and red blood cells (RBC) and without anticoagulant for serum collection. LDL isolation, oxygen radical absorbance capacity (ORAC), AOPP, and malondialdehyde (MDA) measurements were completed using plasma samples, whereas antioxidant enzymes activity and the ratio of reduced glutathione (GSH) to oxidized glutathione (GSSG) levels were evaluated in RBC samples. Serum samples were used to evaluate biochemical parameters and nitric oxide metabolites (NOx) levels. At Days 0 and 7, blood collections were made between 7 a.m. and 9 a.m., after fasting for 12 h.

### 2.3. Carotenoid Preparation and Quantification

Christian Hansen of Brazil (Valinhos, SP, Brazil) provided food grade annatto powder and bixin alkaline extract, which were used in this study. Annatto powder had a high content of bixin (30%) and was used for the bixin treatment. Norbixin powder was obtained from the bixin alkaline extract, through acid precipitation [22] and lyophilization, to obtain a powder formulation containing 42% norbixin. Lycopene powder (10% of lycopene) was obtained from Galena Chemistry and Pharmaceutical Ltda (Campinas, SP, Brazil). The placebo was composed of crude maize starch. Powders were manually encapsulated using an analytical balance and capsules, size 1, where the capsule content was varied based on the weight of the individual (dose = 0.05 mg carotenoid/kg b.w.).

The concentrations of bixin, norbixin, and lycopene powder were checked by spectrophotometry using the respective molar extinction coefficients for bixin (*ɛ*_470nm_ = 2826), norbixin (*ɛ*_453nm_ = 3473), and lycopene (*ɛ*_472nm_ = 3450) [23, 24]. Thereafter, carotenoids of bixin and norbixin powders were identified by high-performance liquid chromatography (HPLC) coupled to a photodiode array detector (PDA). Bixin and norbixin were diluted in the mobile phase, filtered in 0.22 *µ*m pore membrane, and injected (20 *μ*L) in an HPLC-PDA (CBM-20A Prominence, Shimadzu LC) coupled to a Synergi 4 *μ*m hydro RP 80A column (250 × 4.6 mm, Phenomenex, Torrance, USA). Samples were run at 0.9 mL/min using the following gradient: 70% A : 30% B for 10 min, 95% A : 5% B for 10 min, and 70% A : 30% B for 10 min, where mobile phase A was acetonitrile containing 2% formic acid and mobile phase B was water containing 2% formic acid. Compounds were identified by comparing the PDA spectra and peak retention time with authentic standards and literature data. Annatto carotenoids were quantified at 460 nm using standard curves of bixin and norbixin.

### 2.4. Anthropometric Parameters

Body weight, body height, and waist circumference (WC) were determined during the screening of volunteers. Body weight (kg) was measured using a portable digital balance. Body height (meters) was verified in the erect individual, with heels together and head and gluteus in contact with a portable stadiometer. WC was determined below the last rib, according to the *Anthropometric Standardization Reference Manual* [25]. These parameters were accompanied during study intervention but did not show any changes (data not shown).

### 2.5. Biochemical Parameters

Colorimetric kits (Labtest, Lagoa Santa, MG, Brazil) were used to determine serum levels of glucose, total cholesterol (TC), high-density lipoprotein-cholesterol (HDL-C), low-density lipoprotein-cholesterol (LDL-C), very low-density lipoprotein-cholesterol (VLDL-C), triglycerides (TG), AST, ALT, urea, and creatinine in a Labmax 100 semiautomatic analyzer, and hemoglobin levels in a spectrophotometer. Serum VLDL-C was estimated through TG levels, and the Friedewald formula was used to calculate serum LDL-C. The atherogenic index was determined by the ratio between non-HDL-C and HDL-C.

### 2.6. LDL Isolation and *Ex Vivo* Oxidation Induced by Copper

LDL was isolated from the fresh plasma samples by discontinuous density gradient ultracentrifugation [26]. EDTA, sucrose, and KBr were added to avoid LDL aggregation and to form density gradient. LDL particles (yellow/orange band) were collected and dialyzed for 16 h at 4°C in phosphate-buffered saline. The protein content of LDL particles was determined [27], and oxidation susceptibility assays of LDL were performed in sequence, using Cu^2+^ to induce oxidation.

Isolated LDL particles (50 *µ*g protein/mL) were incubated with 10 *µ*M of CuSO_4_ in 10 mM phosphate buffer, pH = 7.4, at 37°C by 10 min, and then the oxidation of the LDL lipid moiety was monitored by assessing the formation of conjugated dienes (CD) at 234 nm during 6.5 h at 37°C, in a microplate reader [28]. Data obtained were used to calculate the lag phase and CD oxidation rate. Lag phase was estimated by the intercept between the time axis and the tangent of the slope of the CD absorbance curve during the propagation phase. The slope of the CD absorbance curve was used to calculate the CD oxidation rate.

The oxidation of the LDL protein moiety was analyzed by the loss of tryptophan (Trp) fluorescence, which decreases due to the oxidation of amino acid residues in apolipoprotein B-100 (ApoB-100). Isolated LDL particles (50 *µ*g protein/mL) were incubated with 3.3 *µ*M CuSO_4_ in 10 mM phosphate buffer, pH = 7.4, at 37°C by 10 min, and thereafter, the loss of Trp fluorescence was monitored (excitation at 282 nm and emission at 331 nm) during 6.5 h at 37°C [29], in a microplate reader. Fluorescence measurements were used to calculate the time necessary to lose 50% of Trp fluorescence (*T*_MAX/2_).

### 2.7. Biomarkers of Oxidative Stress

Lipid peroxidation product, MDA, was evaluated in plasma samples that were derivatized with thiobarbituric acid and then analyzed by HPLC-UV/Vis, using a Zorbax ODS C18 5 *µ*m column (250 × 4.6 mm, Agilent, Santa Clara, USA) [30]. Plasma, serum, and RBC end-point/kinetic UV/VIS and fluorescence spectrophotometry assays were completed in a microplate reader. Plasma AOPP levels were measured at 340 nm [31]. A Griess reaction was performed to evaluate NOx levels, in serum, at 540 nm [32]. Plasma antioxidant capacity was analyzed by the ORAC assay that uses 2,2′-azobis(2-amidinopropane)dihydrochloride (AAPH) as the source of peroxyl radicals and fluorescence spectrophotometry (excitation at 485 nm and emission at 528 nm) [33]. GSH and GSSG were determined in the RBC fraction through an ortho-phthalaldehyde reaction by fluorescence spectrophotometry (emission at 350 nm and excitation at 420 nm), using *N*-ethylmaleimide complexation to prevent GSH from interfering with GSSG measurements [34]. Data were expressed as the GSH/GSSG ratio. The activity of antioxidant enzymes was assessed in RBC samples, and the results were normalized by the hemoglobin (Hb) content. Superoxide dismutase (SOD) activity was assessed by the inhibition of epinephrine autoxidation at 480 nm [35], whereas catalase (CAT) activity was quantified by the decomposition of hydrogen peroxide at 240 nm [36]. Glutathione peroxidase (GPx) and glutathione reductase (GR) were determined at 340 nm, through NADPH oxidation, using specific substrates [37, 38].

### 2.8. Statistical Analysis

The number of participants required for this study (16 subjects) was calculated using a power analysis to detect 25% difference among means, considering a standard deviation of 20% [39–41], alpha error of 0.05, and power of 0.8 (80%), using an one-way analysis of variance (ANOVA) hypothesis test.

Data were expressed as delta values between final and baseline values (Day 7 value–Day 0 value) ± standard error. To verify normality and homogeneity, the data were submitted to the Shapiro–Wilk and Levene tests. The one-way ANOVA and Duncan post hoc test were performed to compare deltas values. The associations between variables were assessed by Pearson's correlation analysis. Differences were considered significant when *p* < 0.1. All statistical analysis was completed using Statistica® version 9.0 software system (StatSoft, Inc., 2004).

## 3. Results

Twenty-one volunteers meet the inclusion criteria for the study, but three were excluded because of health problems (exclusion criteria) or other reasons (Figure 1). Eighteen participants were included in the study, but two participants abandoned the study due to an infections disease or other reasons. Thus, 16 participants completed this randomized, placebo-controlled crossover trial. The volunteers' baseline characteristics are shown in Table 1. Serum glucose levels, lipid profile, markers of renal and hepatic function, anthropometric measurements, and blood pressure were within normal values.

Baseline and final serum biochemical parameters are summarized in Table 2. Daily supplementation with lycopene or annatto carotenoids for one week did not change serum glucose or lipid profile nor did it change the markers of hepatic (AST and ALT) or renal (urea and creatinine) function. Glucose, TC, HDL-C, LDL-C, VLDL-C, TG, AST, ALT, creatinine, and urea levels remained within normal levels after the treatment. Likewise, the atherogenic index of plasma, which is a strong predictor of the risk of atherosclerosis and coronary heart disease, did not show alterations during the study.

**Figure 3:**
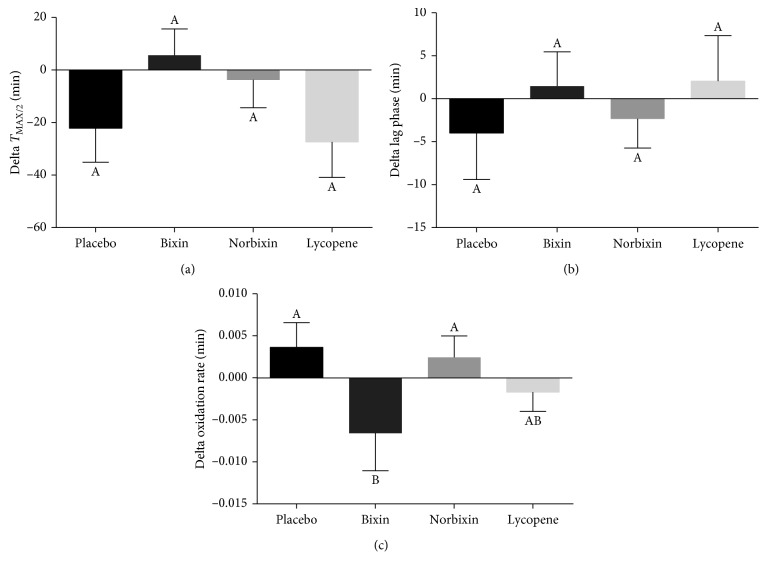
Effect of carotenoid supplementation on the susceptibility of LDL to Cu^2+^-induced oxidation *ex vivo*. The oxidation of the protein moiety was assessed as the time required to lose half-maximal tryptophan fluorescence (a), whereas the oxidation of the lipid moiety was assessed as the lag time for the formation of conjugated dienes (b) and the oxidation rate (c). Results (mean ± SEM) are presented as the delta values between final and baseline values. Different letters indicate a significant difference among treatments (*p* < 0.1).

The oxidation of LDL induced by Cu^2+^*ex vivo* was assessed to determine LDL susceptibility to oxidative insults, which may play a key role in the first steps of atherosclerosis (Figure 3). The oxidation of the LDL protein moiety was assessed as the loss of Trp fluorescence and was expressed as *T*_MAX/2_. CD formation, which characterized the oxidation of the lipid moiety of LDL, was evaluated as the initial lag phase time (Lag phase) and the maximal formation rate of CD (oxidation rate). Greater susceptibility to oxidation is indicated by lower values of *T*_MAX/2_ and lag phase time and higher values of oxidation rate. Dietary supplementation with carotenoids did not affect *T*_MAX/2_ (Figure 3(a)) or lag phase time (Figure 3(b)) values. However, bixin supplementation reduced the CD oxidation rate compared to placebo (Figure 3(c), *p* < 0.1), whereas lycopene and norbixin supplementation had no effect.

**Figure 4:**
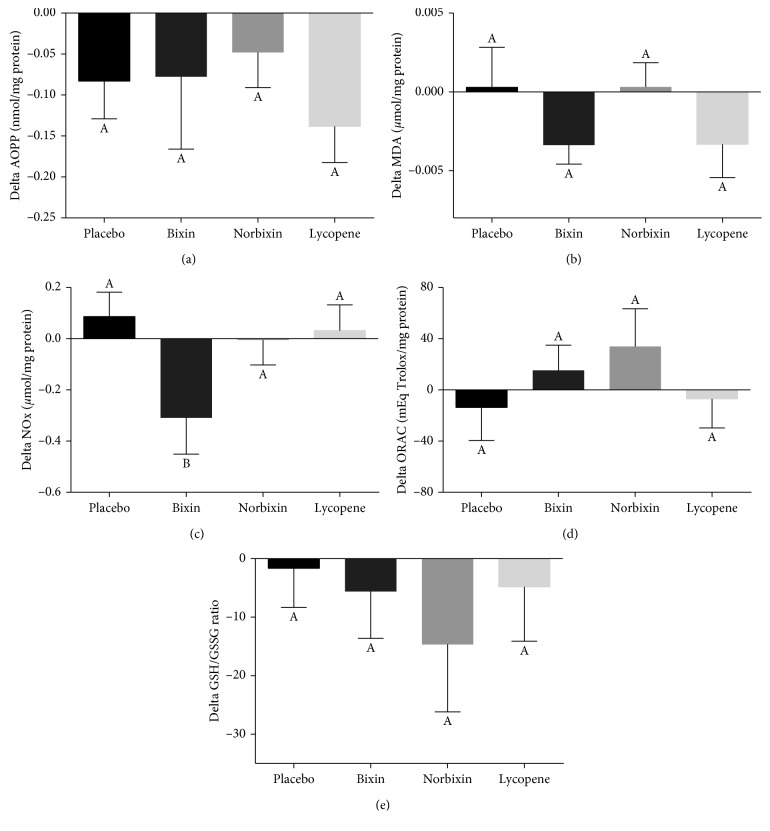
Effect of carotenoid supplementation on plasma protein (a) and lipid (b) oxidation, serum NOx levels (c), plasma antioxidant capacity (d), and red blood cells GSH/GSSG ratio (e). Results (mean ± SEM) are presented as the delta values between final and baseline values. Different letters indicate significant difference among treatments (*p* < 0.1). AOPP: advanced oxidation protein products; MDA: malondialdehyde; NOx: nitric oxide metabolites (nitrite and nitrate); ORAC: oxygen radical absorbance capacity; GSH: reduced glutathione; GSSG: oxidized glutathione.

The oxidative stress status was analyzed by AOPP, MDA, and NOx levels, in addition to GSH/GSSG ratio and ORAC values (Figure 4). Plasma protein oxidation, assessed by AOPP levels (Figure 4(a)), had a slight decrease in all groups, including placebo during the treatment, but no differences were detected among treatments. Carotenoid treatments also did not cause significant changes in plasma lipid oxidation (MDA levels) (Figure 4(b)). Nitric oxide was indirectly assessed by the levels of their metabolites nitrite and nitrate (NOx assay) because it has a short half-life. Bixin supplementation significantly reduced NOx levels compared to the placebo treatment (Figure 4(c), *p*=0.073). MDA and serum antioxidant capacity assessed by ORAC values (Figure 4(d)) and the GSH/GSSG ratio (Figure 4(e)) were not changed by any carotenoid treatment compared to the placebo group.

**Figure 5:**
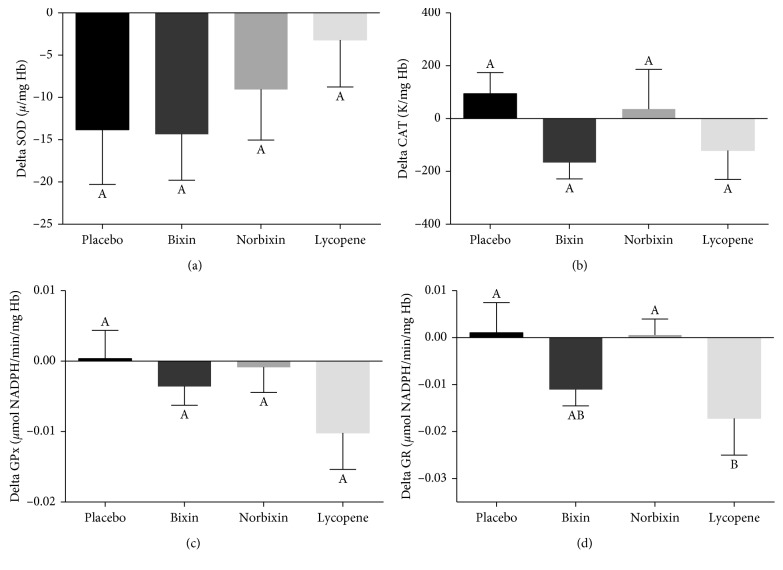
Effect of carotenoid supplementation on superoxide dismutase (a), catalase (b), glutathione peroxidase (c), and glutathione reductase (d) activities in red blood cells. Results (mean ± SEM) are presented as the delta values between final and baseline values. Different letters indicate a significant difference among treatments (*p* < 0.1). SOD: superoxide dismutase; CAT: catalase; GPx: glutathione peroxidase; GR: glutathione reductase.

The activity of antioxidant enzymes from RBC was assessed as a marker of enzymatic antioxidant defenses (Figure 5). RBC SOD activity decreased during the intervention period, but no difference was observed among treatments. RBC CAT and GPx activities also did not differ among treatments. Interestingly, lycopene supplementation reduced RBC GR activity compared to the placebo treatment, whereas bixin and norbixin had no effect.

**Figure 6:**
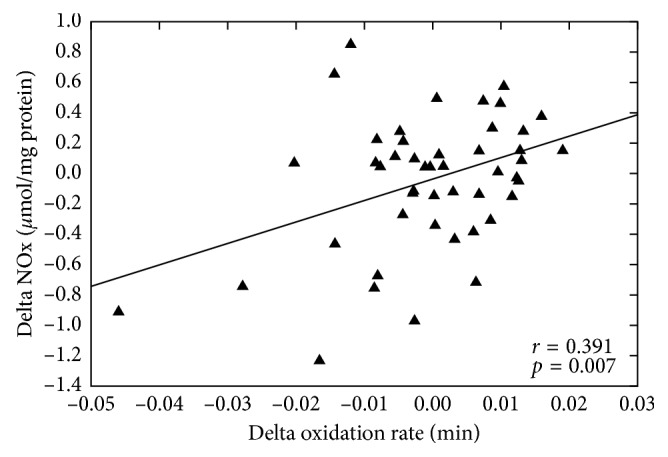
Relationship between serum NOx levels and the oxidation rate of the lipid moiety of LDL particles *ex vivo*. NOx: nitric oxide metabolites (nitrite and nitrate); LDL: low-density lipoprotein.

There was a significant positive relationship between the changes in NOx levels and the CD oxidation rate of LDL particles after treatment (*r* = 0.391, *p*=0.007; Figure 6).

## 4. Discussion

In this study, the effect of short-term, 1-week supplementation with bixin or norbixin was investigated for the first time, regarding the resistance of LDL-C to Cu^2+^-induced oxidative modifications and biochemical and oxidative stress biomarkers in a young healthy population, using lycopene supplementation as a positive control. The short-term supplementation with carotenoids did not modify biochemical biomarkers or most of the oxidative stress markers evaluated. The highlight of our study was the ability of bixin to decrease the oxidation rate of LDL lipid moiety induced by Cu^2+^*ex vivo*, which was parallel to the decrease in NOx levels.

Several studies demonstrated the positive association between dietary intake of lycopene-rich foods and decreased risk of cardiovascular diseases [42]. A recent meta-analysis confirmed that the short-term (1–6 months) intake of tomato products (70–400 g/day) can reduce LDL-C levels, blood pressure, and inflammatory factors [14]. The effect of annatto carotenoids on lipid metabolism has been studied in various animal models. Long-term treatment with bixin has been demonstrated to improve HDL-C levels and decrease atherogenic index in hypercholesterolemic rabbits [18], in addition to reducing LDL-C and TG levels in diabetic rats [17]. In our study, the short-term supplementation with low doses of lycopene or annatto carotenoids was not able to change the serum lipid profile or glycemia. These findings are in accordance with a single-dose study in humans, where bixin and norbixin intake, with a high calorie meal, did not change the postprandial serum lipid profile or glycemia [19]. In the same way, lycopene-enriched tomato sauce consumption for 4 weeks by healthy young subjects also did not improve TC, TG, HDL-C, and glucose levels [43]. In fact, the lipid-lowering effect of tomato products appears to depend on the supplementation time, as a minimum of 4 weeks appears to be required to observe effects in metabolic-syndrome patients, marathon-runners, overweight, and healthy subjects. Moreover, various studies found lipid-lowering effects for lycopene-containing food products that also contained other bioactive compounds, but not for lycopene alone [14].

Although we used short-term supplementation with carotenoids at doses lower than their ADI values, we monitored potential changes in hepatic and renal function during supplementation. As expected, carotenoid treatment did not alter aminotransferases activity or creatinine and urea levels, confirming the safety of this dose.

Hypercholesterolemia, mainly LDL accumulation, has an important role in atherosclerosis development [44]. LDL particles can infiltrate the arterial wall and undergo oxidative modifications in lipids and ApoB100 (ox-LDL), which enables the uptake of ox-LDL particles by macrophages [45]. RS generated by enzymes such as NADPH oxidase, xanthine oxidase, and lipooxygenases can promote LDL oxidation in the vascular wall [4, 46]. Transition metals, such as copper, may also be involved in LDL oxidation *in vivo*, and thus *in vitro* methodologies simulate this condition using Cu^2+^ to induce LDL oxidation [47, 48]. Cu^2+^-induced LDL oxidation comprises an initial phase characterized by antioxidant consumption and minimal lipid peroxidation, which is followed by a propagation phase characterized by extensive peroxidation of polyunsaturated fatty acids and ApoB100 fragmentation [9].

Carotenoids, such as lycopene, astaxanthin, and lutein, have been shown to decrease ox-LDL formation due to its antioxidant action [10]. Tomato juice and ketchup consumption have been demonstrated to increase the resistance of LDL against Cu^2+^-induced oxidation in healthy subjects [49]. However, other studies found no effect of lycopene, demonstrating that lycopene benefits are still unclear [50]. In the present study, lycopene or norbixin supplementation did not improve LDL resistance to Cu^2+^-induced oxidation. However, bixin intake reduced the CD oxidation rate of LDL, although it was not able to attenuate the oxidation of the protein moiety or increase the lag phase for the oxidation of the lipid moiety of LDL.

The resistance of LDL to oxidation in *ex vivo* assays may be influenced by LDL composition [51]. The lag phase for the *ex vivo* oxidation of the lipid moiety is enhanced by LDL-antioxidant content, while the propagation phase can be modified by the content of preformed lipid hydroperoxides (LOOH) [51]. The Cu^2+^-induced oxidation of LDL is dependent on the presence of LOOH, which has an important role in the propagation phase [52]. Therefore, *in vitro* LOOH removal prevents LDL from oxidation induced by Cu^2+^ and other agents [53]. Thus, we believe that the bixin effect on the CD oxidation rate may be related to reduction of LOOH formation in LDL particles during the supplementation period.

LDL peroxidation and, consequently, LOOH formation, is promoted by RS generated by macrophages, endothelial, and smooth muscle cells [54]. Hydrogen peroxide, O_2_^−•^, the hydroxyl radical, and ONOO^−^ are RS involved in this process [46]. ONOO^−^ is a potent oxidant that can be formed by the reaction between NO and O_2_^−•^ at the physiological pH, while in the presence of H^+^, peroxynitrous acid (ONOOH) is generated and can be decomposed yielding products that also have a role in lipid peroxidation [5]. Different mechanisms are involved in O_2_^−•^ production [4], whereas NO is generated by three NO synthase isoforms that use L-arginine as a substrate [55]. eNOS and iNOS have a relationship with cardiovascular pathophysiology, but only iNOS activity is associated with ONOO^−^ generation, oxidative damage, and proatherosclerotic effects [55].

*In vitro* studies have shown that lutein [56], *β*-carotene [57], and astaxanthin [58] inhibit iNOS expression in macrophage cells via downregulation of the NFκB pathway. Reduced expression of iNOS leads to decrease in NO levels [58]. Previous data from our research group has shown that bixin inhibits the protein expression of iNOS in macrophages exposed to oxLDL (Somacal et al., unpublished data) and reduces increased protein oxidation and NOx levels in diabetic rats [17]. In the present study, dietary supplementation with bixin reduced serum NOx levels compared to placebo, whereas norbixin and lycopene had no effect. We also observed a positive correlation between NOx levels and the CD oxidation rate of LDL. Thus, we propose that the increased resistance of LDL to oxidation triggered by bixin supplementation is underlined by the reduction of NOx levels, possibly mediated by the inhibition of iNOS expression. Lower NOx levels are expected to decrease ONOO^−^ and LOOH level in LDL particles.

The control of oxidative stress, either by reduction in RS generation or by its detoxification, is an important event to prevent atherosclerosis development. Beyond the LDL “challenge test” and NOx levels, other oxidative stress biomarkers such as AOPP, MDA, the GSH/GSSG ratio, and ORAC levels can also estimate oxidative stress *in vivo*. Despite the antioxidant capacity of carotenoids, lutein supplementation in new born infants did not change AOPP levels [59], which in addition to being a protein oxidation biomarker, also plays a proinflammatory action [8]. MDA levels reveal the extension of oxidative damage to lipids. Despite evidence that carotenoids can protect lipids from RS, some clinical trials, especially with lycopene, demonstrated that lycopene supplementation is not related to the reduction of MDA levels [60]. While AOPP and MDA levels indicate protein and lipid oxidation, the GSH/GSSG ratio estimates the redox status [61], whose imbalance is associated with early atherosclerosis development [62]. Some carotenoids such as *β*-carotene, astaxanthin, and lutein have been shown to improve cell redox status by increasing GSH levels [63]; however, the effect of lycopene consumption have conflicting results in antioxidant capacity assessments, such as the plasma ORAC assay [64]. In the present study, carotenoid treatment did not affect AOPP, MDA, or ORAC values nor did it affect the GSH/GSSG ratio. Although a previous study demonstrated that a single dose of norbixin reduces the postprandial lipid oxidation triggered by a high calorie meal in humans [19], whereas bixin and norbixin have been shown to attenuate lipid and protein oxidation in animal models [17, 18, 65], this is the first study on the effect of annatto carotenoids on the GSH/GSSG ratio and plasma ORAC.

Antioxidant enzymes such as SOD, CAT, GPx, and GR help endogenous and exogenous nonenzymatic antioxidants to protect against oxidative damage, by converting RS into less reactive species and water or by regenerating endogenous antioxidants, namely, GSH [6]. Animal and human studies have shown that lycopene consumption increases the activity of antioxidant enzymes [63]. In addition, bixin and norbixin increased antioxidant enzymes activity in animal models of diabetes and atherosclerosis [17, 18], while bixin upregulated gene expression of SOD and CAT in a mouse model of diabetic cardiomyopathy [66]. However, only norbixin has increased the postprandial activity of GPx in humans after the concomitant intake with a high calorie meal [19]. The short-term dietary supplementation with lycopene, bixin, or norbixin did not change SOD, CAT, or GPx activities. The interspecies differences and differences in the intervention time and dose may explain the absence of the effect. However, there was a reduction in GR activity after lycopene supplementation, which may be an adaptive response to a lower requirement of antioxidant enzymatic defenses due to the increase in nonenzymatic defenses.

## 5. Conclusion

Short-term oral supplementation of lycopene or norbixin did not affect biochemical parameters or oxidative stress biomarkers in healthy subjects. However, bixin supplementation reduced the oxidation rate of the lipid moiety of LDL particles in a Cu^2+^-induced *ex vivo* assay, probably due to the reduction of NOx levels. Thus, we propose that bixin supplementation could prevent early oxidative modifications in LDL, which is a key event in the development of atherosclerosis.

## Figures and Tables

**Table 1:** Baseline characteristics of 16 volunteers enrolled in the randomized, controlled clinical trial.

Parameters	Population (*n*=16)	Reference values
Age (years)	25.4 ± 0.8	—
Weight (kg)	66.5 ± 2.2	—
Height (m)	1.71 ± 0.03	—
Sex	8 males/8 females	—
Waist circumference (cm)	80.9 ± 2.1	Men < 94.0
		Women < 80.0
Systolic blood pressure (mmHg)	110 ± 0.5	<120
Diastolic blood pressure (mmHg)	80 ± 0.4	<80
Body mass index (kg/cm^2^)	22.6 ± 0.7	18.5–24.9
ALT (U/L)	19.0 ± 2.0	<45.0
AST (U/L)	18.7 ± 1.6	<35.0
TC (mg/dL)	179.6 ± 6.9	<190.0
HDL-C (mg/dL)	52.0 ± 4.3	>40.0
LDL-C (mg/dL)	110.1 ± 4.9	<130.0
TG (mg/dL)	87.4 ± 12.4	<150.0
Glucose (mg/dL)	92.5 ± 2.4	60.0–100.0
Creatinine (mg/dL)	1.15 ± 0.06	0.4–1.4
Urea (mg/dL)	28.7 ± 1.7	10–50.0

Values are expressed as mean ± SEM. ALT, alanine aminotransferase; AST, aspartate aminotransferase; TC, total cholesterol; HDL, high-density lipoprotein; LDL, low-density lipoprotein; TG, triglycerides.

**Table 2:** Serum biochemical parameters before (Day 0) and after (Day 7) oral supplementation of carotenoids.

Parameters	Placebo			Bixin			Norbixin			Lycopene		
	Day 0	Day 7	Change	Day 0	Day 7	Change	Day 0	Day 7	Change	Day 0	Day 7	Change
Glucose (mg/dL)	83.7 ± 2.2	81.9 ± 1.8	−1.8 ± 2.1	84.5 ± 2.9	84.4 ± 3.5	−0.1 ± 1.3	82.7 ± 2.4	85.6 ± 2.4	2.9 ± 2.1	84.6 ± 3.0	84.5 ± 2.4	−0.1 ± 1.7
TC (mg/dL)	176.4 ± 8.7	177.3 ± 6.7	0.9 ± 5.2	180.8 ± 8.8	181.1 ± 10.1	0.3 ± 4.6	174.8 ± 11.7	179.3 ± 1.0	4.5 ± 5.9	176.7 ± 8.2	185.6 ± 8.8	8.9 ± 4.1
HDL-C (mg/dL)	51.5 ± 3.9	49.6 ± 4.2	−1.9 ± 1.9	49.7 ± 3.1	50.9 ± 3.6	1.2 ± 1.81	52.1 ± 4.6	53.1 ± 5.5	1.0 ± 2.7	53.6 ± 3.0	54.1 ± 3.3	0.5 ± 2.5
LDL-C (mg/dL)	110.7 ± 7.1	109.3 ± 6.4	−1.4 ± 4.8	113.6 ± 6.8	118.4 ± 9.6	4.8 ± 7.2	101.6 ± 6.6	106.4 ± 6.4	4.8 ± 5.8	109.7 ± 6.5	114.8 ± 6.8	5.1 ± 5.6
TG (mg/dL)	88.1 ± 12.3	99.5 ± 12.4	11.4 ± 5.6	93.4 ± 12.5	102.6 ± 11.5	9.2 ± 7.1	93.4 ± 11.6	99.2 ± 12.8	5.8 ± 4.5	81.5 ± 10.6	86.7 ± 9.2	5.2 ± 5.5
VLDL-C (mg/dL)	17.8 ± 2.6	19.4 ± 2.6	1.6 ± 1.1	18.7 ± 2.5	20.5 ± 2.3	1.8 ± 1.4	18.7 ± 2.3	19.8 ± 2.5	1.1 ± 0.9	16.3 ± 2.1	17.3 ± 1.8	1.0 ± 1.1
AST (U/L)	15.2 ± 1.0	15.4 ± 1.1	0.2 ± 0.8	16.3 ± 0.9	15.5 ± 1.1	−0.8 ± 0.7	13.7 ± 0.9	14.6 ± 0.7	0.9 ± 0.6	15.4 ± 0.9	16.5 ± 0.8	1.1 ± 0.8
ALT (U/L)	15.4 ± 2.1	15.0 ± 1.7	−0.4 ± 1.0	18.7 ± 1.8	17.5 ± 2.2	−1.1 ± 1.5	13.9 ± 1.6	12.7 ± 1.2	−1.2 ± 1.2	16.4 ± 1.5	17.3 ± 1.5	0.9. ± 0.9
Creatinine (mg/dL)	1.17 ± 0.06	1.14 ± 0.07	−0.03 ± 0.05	1.09 ± 0.06	1.11 ± 0.05	0.02 ± 0.03	1.09 ± 0.05	1.05 ± 0.04	−0.04 ± 0.04	1.1 ± 0.1	1.14 ± 0.09	0.04 ± 0.05
Urea (mg/dL)	23.4 ± 1.5	26.1 ± 1.7	2.7 ± 0.9	26.9 ± 1.6	26.0 ± 1.7	−0.9 ± 1.4	27.1 ± 1.6	27.4 ± 1.8	0.3 ± 1.4	28.2 ± 1.3	26.2 ± 1.7	−2.0 ± 1.1
Atherogenic index	2.75 ± 0.24	2.91 ± 0.37	0.16 ± 0.18	2.72 ± 0.2	2.72 ± 0.21	0.0 ± 0.2	2.67 ± 0.3	2.52 ± 0.3	−0.14 ± 0.23	2.5 ± 0.2	2.56 ± 0.21	0.06 ± 0.2

Data are expressed as means ± SEM of 16 volunteers. TC: total cholesterol; HDL-C: high-density lipoprotein-cholesterol; LDL-C: low-density lipoprotein-cholesterol; TG: triglycerides; VLDL-C: very low-density lipoprotein-cholesterol; AST: aspartate aminotransferase; ALT: alanine aminotransferase. Change = Day 7–Day 0.

## Data Availability

The data used to support the findings of this study are available from the corresponding author upon request.
